# The metaverse in nuclear medicine: transformative applications, challenges, and future directions

**DOI:** 10.3389/fmed.2024.1459701

**Published:** 2024-09-20

**Authors:** Yufu Tang, Hongying Liang, Xin Yang, Xiangming Xue, Jingming Zhan

**Affiliations:** Division of Radiology and Environmental Medicine, China Institute for Radiation Protection, Taiyuan, China

**Keywords:** metaverse, nuclear medicine, virtual reality, augmented reality, artificial intelligence, personalized dosimetry

## Abstract

The metaverse, a rapidly evolving virtual reality space, holds immense potential to revolutionize nuclear medicine by enhancing education, training, diagnostics, and therapeutics. This review explores the transformative applications of the metaverse in nuclear medicine, where immersive virtual learning environments, simulation-based training, artificial intelligence (AI)-powered decision support systems integrated into interactive three-dimensional (3D) visualizations, and personalized dosimetry using realistic patient-specific virtual models are seamlessly incorporated into the metaverse ecosystem, creating a synergistic platform for healthcare professionals and patients alike. However, the responsible and sustainable adoption of the metaverse in nuclear medicine requires a multidisciplinary approach to address challenges related to standardization, accessibility, data security, and ethical concerns. The formation of cross-disciplinary consortia, increased research and development (R&D) investment, and the strengthening of data governance and cybersecurity measures are crucial steps in ensuring the safe and effective integration of the metaverse in healthcare. As the metaverse continues to evolve, researchers, practitioners, and policymakers must collaborate and explore its potential, navigate the challenges, and shape a future where technology and medicine seamlessly integrate to enhance patient care and outcomes in nuclear medicine. Further research is needed to fully understand the implications of the metaverse in clinical practice, education, and research, as well as to develop evidence-based guidelines for its responsible implementation. By embracing responsible innovation and collaboration, the nuclear medicine community can harness the power of the metaverse to transform and improve patient care.

## 1 Introduction

The metaverse, a digital shared space built upon the foundations of virtual reality (VR) and augmented reality (AR), is further enhanced by integrating cutting-edge technologies such as artificial intelligence (AI) and blockchain, creating a powerful combination that has the potential to revolutionize various industries. The term “metaverse” was first coined by Neal Stephenson in his 1992 science fiction novel “Snow Crash,” where it was described as a virtual world that exists parallel to the real world, constantly online and influenced by real-world events (1), and is revolutionizing various sectors. In education, the metaverse enables immersive learning experiences, allowing students to interact with virtual objects and simulations, enhancing engagement and knowledge retention (2). The entertainment industry has also embraced the metaverse, creating interactive and personalized user experiences (3). Moreover, the metaverse has found applications in tourism, enabling virtual tours and experiences, and in the automotive industry, facilitating virtual vehicle design and testing (4, 5).

The metaverse has also found notable applications in other medical and health fields. For instance, in orthopedics, the metaverse has been used to create immersive virtual environments for surgical training and planning, allowing surgeons to practice complex procedures risk-free (6). In mental health, metaverse-based interventions have shown promise in delivering engaging and effective therapy, such as exposure therapy for anxiety disorders (7). Furthermore, the metaverse has been explored as a platform for remote patient monitoring and telemedicine, enabling healthcare providers to deliver personalized care and support to patients in virtual environments (8).

Nuclear medicine is a critical medical field that uses radioactive isotopes for diagnosing, treating, and researching diseases in various specialties, such as oncology, cardiology, and neurology (9). Advanced imaging techniques, including single-photon emission computed tomography (SPECT) and positron emission tomography (PET), provide functional information that helps detect diseases early, assess treatment efficacy, and guide clinical decisions (10, 11). However, nuclear medicine has limitations. The primary concern is radiation exposure risk during procedures, requiring appropriate protective measures for medical staff and patients (12). Additionally, nuclear medicine imaging has lower spatial resolution than other modalities, potentially impacting the detection of small lesions (13). Nuclear medicine education and training often rely on two-dimensional (2D) images and simplistic models, limiting students’ understanding of complex anatomical structures and physiological processes (14).

The integration of the metaverse into nuclear medicine has the potential to address these limitations and enhance various aspects of the field. By leveraging VR, AR, and mixed reality (MR) technologies, the metaverse can provide immersive and interactive visualizations of three-dimensional (3D) medical images, enabling more precise diagnostics and treatment planning (15). Metaverse-based simulations can also improve nuclear medicine education and training, allowing students to interact with virtual radiopharmaceuticals, practice procedures, and explore anatomical structures in a risk-free environment (16). Furthermore, the metaverse can facilitate remote collaboration among healthcare professionals, enabling expert consultations and knowledge sharing across geographical boundaries (17).

This review aims to explore the transformative potential of the metaverse in nuclear medicine, focusing on its applications, challenges, and future directions. By examining how the metaverse can enhance education, training, diagnostics, and therapeutics in nuclear medicine, we seek to inform researchers, practitioners, and policymakers about the opportunities and challenges of this emerging technology. As the metaverse continues to evolve, it is crucial to understand its potential impact on nuclear medicine and healthcare, shaping a future where technology and medicine seamlessly integrate to improve patient care and outcomes.

## 2 Methods

To identify relevant papers and applications, a comprehensive literature search was conducted using the following databases: PubMed, Scopus, IEEE Xplore, and Web of Science. The search terms included combinations of “metaverse,” “nuclear medicine,” “virtual reality,” “augmented reality,” “mixed reality,” “education,” “training,” “diagnostics,” “therapeutics,” “PET,” “SPECT,” “radiotherapy,” and “radiotheranostics.” The search was limited to articles published in English between 2010 and 2024. Additionally, a manual search of the reference lists of the retrieved articles was performed to identify any additional relevant studies. The inclusion criteria were as follows: (1) studies focusing on the application of metaverse technologies in medicine and health, (2) original research articles, review papers, and case reports, and (3) studies providing sufficient information on the design, implementation, and evaluation of metaverse applications. Exclusion criteria were: (1) non-English publications, (2) studies not directly related to the metaverse in nuclear medicine, and (3) abstracts, editorials, and conference proceedings.

## 3 Metaverse applications in nuclear medicine education and training

Several studies have highlighted the potential of virtual and augmented reality in medical education, which has demonstrated improved knowledge acquisition, skill development, and learner satisfaction compared to traditional teaching methods (18, 19). With its immersive and interactive capabilities, the metaverse can enhance the learning experience and outcomes in nuclear medicine education by providing unique opportunities for experiential learning and realistic simulations (16, 20).

### 3.1 Immersive virtual learning and simulation training platforms

In the metaverse, aligning intended learning outcomes in the cognitive, affective, and psychomotor domains with learning and simulation mechanics is a promising instructional design approach (21). For instance, a study on the impact of visual complexity of animated virtual agents (AVAs) on learning outcomes in virtual reality learning applications demonstrated that highly realistic AVAs can increase learner engagement and learning performance (22). Immersive virtual learning environments in the metaverse can provide a highly realistic and interactive platform for students to explore and manipulate complex nuclear medicine scenarios. These environments allow students to visualize and interact with 3D models of anatomical structures, radiopharmaceuticals, and imaging equipment, enhancing their understanding of fundamental concepts and ability to apply them in real-world situations.

The virtual radiation laboratory showcases VR’s potential in creating realistic scenarios that allow nuclear medicine professionals to interact with virtual radiation sources and practice safety procedures (23). By providing a safe and controlled learning environment, students can deeply understand radiation protection principles and gain practical skills without the risks of handling actual radioactive materials.

The Virtual Environments for Radiotherapy Training (VERT) system (24) is an exemplary model of the potential of immersive virtual learning. VERT enables students to practice radiotherapy planning and delivery in a realistic setting, providing a safe space for experimentation and problem-solving. Through this immersive experience, students can develop critical spatial awareness and decision-making skills, preparing them for the challenges they will face in clinical practice.

Moreover, experienced nuclear medicine professionals, including senior physicians and technologists, can also leverage these technologies to practice and experience rare or complex cases in realistic virtual environments, enhancing their ability to manage challenging clinical situations (25). Metaverse platforms provide a collaborative space for knowledge sharing among professionals across institutions and geographical boundaries, facilitating the exchange of expertise and promoting continuous learning. Regular simulation training and case discussions in virtual environments enable seasoned nuclear medicine professionals to stay abreast of the latest knowledge and skills, elevating their professional competence (26).

### 3.2 Augmented reality exploration and radiopharmaceutical preparation simulation training

The AR technology has shown great potential in various medical fields, including surgical procedures and rehabilitation. For instance, AR can superimpose patient information and surgical guidance onto the surgeon’s field of view during procedures, enhancing precision and reducing errors (27). Additionally, AR has been used as a distraction technique in pain management and as a therapy for mental health conditions such as anxiety, depression, and phobias (28).

The AR learning modules in the metaverse offer a new dimension to nuclear medicine education by providing real-time, interactive visualizations of complex anatomical structures, physiological processes, and medical imaging data. The HoloLens-based AR system, HoloAnatomy (29), demonstrates the power of AR in teaching complex anatomical concepts. This system allows students to interact with 3D holographic models and explore the human body in unprecedented detail, enhancing their comprehension and retention of the subject matter.

Simulation-based training in the metaverse provides a game-changing approach to teaching radiopharmaceutical preparation and handling. By leveraging VR and AR, nuclear medicine technologists can practice these essential skills without the risks of handling actual radioactive materials. Immersing learners in a virtual radiopharmacy environment enables them to develop muscle memory and confidence in their abilities, ultimately improving performance and patient safety when transitioning to clinical settings (30, 31).

## 4 Metaverse-enabled advancements in nuclear medicine diagnostics

The metaverse is poised to profoundly impact nuclear medicine diagnostics: 3D visualization and image fusion, telemedicine and multidisciplinary collaboration, and AI-powered diagnostic and decision support tools. By examining the current state of these technologies and their potential applications within the metaverse, we aim to provide a comprehensive overview of the paradigm shift underway in nuclear medicine diagnostics. This exploration will shed light on how the metaverse can empower nuclear medicine professionals to push the boundaries of what is possible in disease detection, characterization, and treatment planning, ushering in a new era of personalized medicine and improved patient care.

### 4.1 3D visualization and image fusion

Current evidence-based medicine suggests that PET/computed tomography (CT) is significant in patient selection, treatment response evaluation, and prognostic prediction. Although most evidence comes from retrospective studies, PET/CT has shown promising potential in this field (32–34). 3D visualization and image fusion in the metaverse provide a highly immersive and interactive environment for analyzing complex nuclear medicine imaging data. Integrating PET/SPECT with CT/magnetic resonance imaging (MRI) in a virtual 3D space allows a more comprehensive understanding of disease processes. This enables clinicians to identify subtle lesions and characterize them with greater precision. The Virtual 3D PET/CT system (35) exemplifies this concept, providing a platform for clinicians to interact with fused PET/CT images in a highly intuitive and flexible manner. This system enhances diagnostic accuracy and facilitates more effective communication between nuclear medicine professionals and referring physicians, ultimately improving patient care.

### 4.2 Telemedicine and multidisciplinary collaboration

Telemedicine and multidisciplinary collaboration in the metaverse break down geographical barriers, enabling experts from different specialties and locations to collaborate seamlessly in a virtual environment (36, 37). The Virtual Tumor Board (VTB) platform (38) demonstrates the transformative potential of VR in facilitating multidisciplinary cancer case discussions. By bringing together oncologists, radiologists, pathologists, and other specialists in a shared virtual space, the VTB platform enables more informed and comprehensive treatment decision-making. This collaborative approach improves patient outcomes and fosters knowledge sharing and learning among healthcare professionals, ultimately elevating the standard of care in nuclear medicine.

### 4.3 AI-powered diagnostic and decision support

AI-powered diagnostic and decision support tools in the metaverse offer a new frontier in personalized medicine, providing real-time assistance and treatment recommendations tailored to individual patients. Hong et al. (39) enhance PET image quality, reduce costs, and potentially lower radiation doses, coupled with the integration of AI for automated lesion detection and characterization, exemplifies the transformative potential of these technologies in revolutionizing nuclear medicine diagnostics and personalized patient care (13). The AI enhances diagnostic efficiency and enables clinicians to explore and interact with imaging data in novel ways, potentially uncovering new insights and improving patient outcomes.

## 5 Challenges and considerations for metaverse adoption in nuclear medicine

While offering tremendous potential for innovation and advancement, adopting metaverse technologies in nuclear medicine presents complex challenges and considerations that must be carefully addressed to ensure responsible and sustainable implementation. These challenges span technical, economic, and ethical domains, requiring a concerted effort from researchers, practitioners, policymakers, and industry stakeholders to navigate the path forward.

### 5.1 Standardization and interoperability challenges

Standardization and interoperability represent a critical technical challenge in integrating metaverse technologies within the nuclear medicine ecosystem. The need for standardized protocols and data formats can create significant barriers to the seamless exchange of information between various VR/AR systems and medical devices, hindering the development of cohesive and efficient workflows. The Medical Extended Reality (MXR) Interoperability Framework proposed by the IEEE (40) represents a crucial step toward addressing this challenge, providing a comprehensive set of standardized data exchange and communication guidelines. By establishing a common language and framework for interoperability, the MXR Interoperability Framework can enable the development of more integrated and user-friendly metaverse solutions that can be readily adopted across different healthcare settings.

### 5.2 Accessibility and cost-effectiveness considerations

Accessibility and cost-effectiveness pose significant economic challenges to adopting metaverse technologies in nuclear medicine. The high cost of advanced VR/AR systems can create a digital divide, limiting access to these transformative technologies for smaller healthcare facilities and underserved communities. To address this challenge, there is a pressing need to develop more affordable and user-friendly metaverse solutions that can democratize access to these cutting-edge tools. The MedVR system, a low-cost VR platform for medical education, represents a promising example of how innovative design and engineering approaches can help bridge the accessibility gap. By leveraging open-source software and off-the-shelf hardware components, MedVR demonstrates the potential for creating cost-effective and scalable metaverse solutions that many healthcare institutions can readily adopt.

### 5.3 Data security and ethical concerns

Data security and ethical concerns represent the most complex and multifaceted challenges of adopting metaverse technologies in nuclear medicine. Collecting, storing, and sharing sensitive patient data within virtual environments raises critical questions about privacy, confidentiality, and informed consent (41). Lee et al. (42) introduced a blockchain-based decentralized patient information exchange system that ensures the secure and efficient sharing of electronic medical records while protecting patient privacy. Simulation results and security analysis demonstrate that the system effectively safeguards against security threats such as data forgery and privacy leaks, ensures data integrity, and supports rapid sharing of electronic medical records of various sizes and formats. This study highlights the potential of blockchain technology in facilitating patient-centric health information exchange and access control. However, implementing such systems raises critical ethical questions about data ownership, user autonomy, and potential unintended consequences, such as exacerbating health disparities.

### 5.4 Multidisciplinary collaboration and stakeholder engagement

Navigating these challenges and considerations will require a multidisciplinary and collaborative approach that brings together expertise from nuclear medicine, computer science, bioethics, and public policy (43). Fostering ongoing dialog and engagement among these diverse stakeholders will be essential to developing robust standards, guidelines, and best practices for the responsible adoption of metaverse technologies in healthcare. This process must be grounded in a shared commitment to patient-centered care, health equity, and the ethical stewardship of sensitive medical data.

## 6 Future directions and recommendations

Future directions and recommendations should be considered to fully harness the metaverse’s transformative potential in nuclear medicine. These include the formation of cross-disciplinary consortia for standardization, increased research and development (R&D) investment in metaverse technologies, and strengthening data governance and cybersecurity measures.

### 6.1 Fostering cross-disciplinary collaboration for standardization

Cross-disciplinary consortia involving experts from nuclear medicine, computer science, engineering, and other relevant fields are essential for establishing standardized protocols and guidelines for metaverse applications in healthcare. The American Medical Extended Reality Association (AMXRA) exemplifies such collaborative efforts. As a non-profit organization dedicated to advancing the application of extended reality (XR) technologies in healthcare, AMXRA brings together experts from academia, industry, and medical institutions (44). Through workshops, working groups, and conferences, AMXRA facilitates cross-disciplinary knowledge sharing, challenge identification, and the development of standards for applying XR technologies in medical education, research, and clinical practice. By fostering cross-disciplinary collaboration and knowledge sharing, these consortia can accelerate the development and adoption of interoperable metaverse solutions that can be readily integrated into existing healthcare workflows.

### 6.2 Driving innovation through increased R&D investment

Increased R&D investment in metaverse technologies is crucial for driving innovation and improving the accessibility, affordability, and user-friendliness of VR/AR systems in nuclear medicine (45). Governments, industry partners, and academic institutions should collaborate to fund and support research initiatives exploring the metaverse’s potential in enhancing diagnostics, therapeutics, and education in nuclear medicine. This investment should focus on developing novel hardware and software solutions that can deliver immersive, interactive, and personalized experiences while also addressing the unique challenges and requirements of the healthcare setting, such as infection control and patient safety.

### 6.3 Strengthening data governance and cybersecurity measures

Strengthening data governance and cybersecurity measures is essential for protecting patient privacy and ensuring the secure management of sensitive medical data in the metaverse (46). A decentralized marketplace for patient-generated health data, leveraging blockchain technology such as Ethereum smart contracts and distributed storage systems like the InterPlanetary File System (IPFS), can significantly enhance data governance and cybersecurity measures within the metaverse (47). This approach ensures data provenance, accuracy, security, and privacy by facilitating secure storage, encryption, and communication between users and the marketplace. The decentralized nature of the marketplace mitigates the risk of data breaches and unauthorized access while providing an immutable record of data transactions for verifiability and transparency. By adopting this approach, the metaverse ecosystem can enable the secure and privacy-preserving sharing of patient-generated health data, ultimately leading to improved healthcare outcomes and research. However, implementing such frameworks must also be accompanied by robust cybersecurity measures, such as encryption, authentication, and access control, to safeguard against potential threats and vulnerabilities.

### 6.4 Prioritizing user-centered design and usability

It is essential to prioritize user-centered design and usability to ensure the successful adoption and integration of metaverse technologies in nuclear medicine. Metaverse solutions should be designed to meet healthcare professionals’ and patients’ needs and preferences, providing intuitive and seamless user experiences that can be easily integrated into existing workflows. This May involve conducting extensive user research, usability testing, and iterative design processes to ensure that metaverse applications are functional, user-friendly, and accessible to a wide range of users, including those with varying technical expertise and physical abilities (48).

### 6.5 Promoting education and training in metaverse technologies

To fully realize the potential of the metaverse in nuclear medicine, it is crucial to promote education and training in metaverse technologies among healthcare professionals, researchers, and students. This may involve developing specialized curricula and training programs focusing on applying VR/AR technologies in healthcare and providing hands-on learning opportunities and simulations that allow users to develop practical skills and experience using metaverse tools (49). Investing in education and training can build a well-equipped workforce to leverage the metaverse’s power in advancing nuclear medicine research, practice, and patient care.

## 7 Conclusion

The metaverse has the potential to revolutionize nuclear medicine by offering immersive, interactive, and realistic experiences that enhance education, diagnostics, and treatment planning. This review highlights the transformative applications of the metaverse in nuclear medicine, including virtual learning environments, simulation-based training, AI-powered decision support, and personalized dosimetry. However, responsible adoption requires a multidisciplinary approach to address challenges related to standardization, accessibility, data security, and ethics.

As the metaverse evolves, further research is needed to understand its implications and develop evidence-based guidelines for implementation. Integrating the metaverse in nuclear medicine could lead to more precise, efficient, and patient-centered care. Researchers, practitioners, and policymakers must collaborate to explore the metaverse’s potential, navigate challenges, and shape a future where technology and medicine seamlessly integrate to enhance patient outcomes. By embracing responsible innovation and collaboration, the nuclear medicine community can harness the metaverse’s power to usher in a new era of precision, personalization, and patient-centered care.
